# IMPLEMENTING GRIEF SERVICES FOR OLDER ADULTS: TRANSLATING SURVEY FINDINGS INTO TARGETED PROGRAMMING

**DOI:** 10.1093/geroni/igad104.1785

**Published:** 2023-12-21

**Authors:** Rachel Weiskittle

**Affiliations:** University of Colorado Colorado Springs, Colorado Springs, Colorado, United States

## Abstract

This presentation will identify and describe the team’s ongoing efforts in actuating the Implementation phase of the EPIS Framework. Evidence-based strategies include formulating a multidisciplinary implementation team, organizing purveyor and intermediary support, and developing a Monitoring, Evaluation, and Learning (MEL) plan. We will highlight the ways in which survey findings were disseminated to the general public and organizational leaders, such as presenting result summaries at city council meetings and in local newspaper articles. The challenges of negotiating survey findings into cohesive program implementation will be discussed, as well as management strategies towards age-related gaps in the implementation process that had not been identified previously. Recent program launches and the ways in which survey results directly informed their delivery will also be reviewed, namely, a free educational guest-speaker event for which over 700 community members attended and a grief-support holiday vigil hosted at a local long-term care facility. This presentation will also review considerations for the project’s next steps as it continues to hone its program delivery parameters and eventually enters into the last EPIS phase, Sustainment. Specific foci will include deciding the long-term methods of quality assurance evaluation and program adaptations to revolving community bereavement needs.

